# MULTI-OMICS INTEGRATION IDENTIFIES GENES INFLUENCING TRAITS ASSOCIATED WITH CARDIOVASCULAR RISKS

**DOI:** 10.1093/geroni/igad104.3482

**Published:** 2023-12-21

**Authors:** Sandeep Acharya, Shu Liao, Woo Seok Jung, Edward Kang, Vaha Akbary Moghaddam, Mary Feitosa, Michael Province, Michael Brent

**Affiliations:** Washington University in St. Louis, St. Louis, Missouri, United States; Washington University in St. Louis, St. Louis, Missouri, United States; Washington University in St. Louis, St. Louis, Missouri, United States; Washington University in St. Louis, St. Louis, Missouri, United States; Washington University in St. Louis, St. Louis, Missouri, United States; Washington University School of Medicine, St. Louis, Missouri, United States; Washington University School of Medicine, St. Louis, Missouri, United States; Washington University in St. Louis, St. Louis, Missouri, United States

## Abstract

The Long Life Family Study (LLFS) enrolled 4,953 participants in 539 pedigrees displaying exceptional longevity. To identify genetic mechanisms that protect LLFS participants against age-related cardiovascular risks, we developed a freely available multi-omics integration pipeline and applied it to 11 traits associated with cardiovascular risks. Using our pipeline, we aggregated gene-level statistics from Rare-Variant Analysis, GWAS, and gene expression-trait association by Correlated Meta-Analysis (CMA). Across all traits, CMA identified 51 significant genes after Bonferroni correction (P ≤ 2.8×10-7). CETP, NLRC5, SLC45A3, and TOMM40 lie within 50 Kb of a known trait-associated variant (previously associated genes). Analysis of protein-protein interaction (PPI) networks identified another 63 genes (passing genes) that (1) have CMA p-value ≤ 5×10-3, (2) lie in a PPI module (highly connected subnetwork) enriched for genes with low P-values, and (3) are annotated with a biological process that is enriched among module genes, ten of which were previously associated with the same traits. Permutation analysis showed that passing genes have a false positive rate of 1 in 14876 and are more likely to be previously known than non-passing genes with similar p-values. CMA improved on the 3 input analyses by producing the largest number of modules enriched for genes with low P-values and highly enriched for genes participating in shared biological processes. Overall, module analysis identified highly plausible candidate causal genes whose P-values after CMA alone were merely suggestive.

